# Insulin Resistance Among Patients With Multiple Endocrine Neoplasia Type 1: A Systemic Review and Meta-Analysis

**DOI:** 10.1016/j.aed.2025.08.018

**Published:** 2025-09-03

**Authors:** Berk Celik, Leticia De Mattei, Julia Meireles, Ayed Shahatit, Maya Kardouh, Lynda Misra, Michael Brennan

**Affiliations:** 1 Department of Internal Medicine, Corewell Health William Beaumont University Hospital, Royal Oak, Michigan; 2 Oakland University William Beaumont School of Medicine, Rochester, Michigan; 3 Division of Endocrinology, Department of Medicine, Corewell Health William Beaumont University Hospital, Royal Oak, Michigan

**Keywords:** insulin resistance, type 2 diabetes mellitus, multiple endocrine neoplasia type 1, MEN1

## Abstract

**Background/Objective:**

Multiple endocrine neoplasia type 1 (MEN1) is a hereditary syndrome characterized by predisposition to tumors of parathyroid, pituitary, and enteropancreatic cells. Multiple studies previously showed increased prevalence of insulin resistance and incidence of cardiovascular diseases, decreased life expectancy in patients with MEN1. In this meta-analysis, we aim to further evaluate the extent of insulin resistance among individuals with MEN1.

**Methods:**

We systematically searched PubMed, Embase, Scopus, Web of Science Core Collection, and the Cochrane Central Register of Controlled Trials (CENTRAL). Studies involving at least 5 patients with MEN1 and reporting on insulin resistance were included. Primary outcome designated as prevalence of insulin resistance. Meta-analyses done and odds ratios were calculated via the random effects model.

**Results:**

Out of 1453 studies, 5 met the inclusion criteria, involving 606 patients with MEN1 and 4431 controls were included in the meta-analysis. Patients with MEN1 had a significantly higher prevalence of insulin resistance with an odds ratio of 5.58 (95% confidence interval [4.02, 7.74]. No heterogeneity was observed (Tau^2^ = 0, *P* = 0.4552). Sensitivity analyses confirmed the robustness of findings.

**Conclusions:**

To our best knowledge, this represents the first meta-analysis on MEN1 and insulin resistance. We have identified a higher prevalence of insulin resistance among individuals with MEN1 compared to controls, independent of age, sex, or body mass index. Given these findings, we recommend that patients with MEN1 should be screened early in the disease course for impaired glucose tolerance, as this may help prevent future cardiovascular events and enhance life expectancy.

**Highlights**

• Patients with multiple endocrine neoplasia type 1 (MEN1) have a significantly higher prevalence of insulin resistance compared to controls, with an odds ratio of 5.58
• Life expectancy in MEN1 is reduced compared to the general population, with cardiovascular disease accounting for up to 20% of non-MEN1-related deaths
• Menin, the protein encoded by the MEN1 tumor suppressor gene, is a co-activator for peroxisome proliferator-activated receptor gamma (PPARγ), a key regulator of glucose and lipid metabolism
• Dysregulation of menin-mediated pathways may contribute to insulin resistance in patients with MEN1 via altered PPARγ activity, impaired hepatic glucose uptake, and disrupted insulin secretion

**Clinical Relevance**

Our study shows that patients with multiple endocrine neoplasia type 1 exhibit a higher prevalence of insulin resistance compared to controls. This metabolic disturbance is likely a key contributor to the increased cardiovascular mortality observed in this patient group. Early screening for insulin resistance may help mitigate long-term cardiovascular risks and improve life expectancy.

## Introduction

Multiple endocrine neoplasia type 1 (MEN1) is an autosomal-dominant hereditary syndrome characterized by predisposition to tumors of parathyroid, pituitary, and enteropancreatic cells.^1^ The disease results from mutations in the MEN1 tumor suppressor gene, which encodes menin, a 610-amino acid nuclear protein. Menin participates in a variety of cellular processes, including transcriptional regulation, maintenance of chromosomal integrity, and deoxyribonucleic acid replication.^2^ Several studies have also demonstrated that menin modulates the transcription of genes involved in insulin sensitivity, functioning as a co-activator for peroxisome proliferator-activated receptor gamma.^3^, ^4^, ^5^

MEN1 exhibits high penetrance, with nearly 100% of mutation carriers developing clinical manifestations by the age of 50.^6^ Primary hyperparathyroidism is the most common feature of MEN1, occurring in virtually all affected individuals, followed by enteropancreatic neuroendocrine tumors in approximately 40% and pituitary adenomas in about 30%.^6^ MEN1 is considered rare, with an estimated incidence of 1 in 30 000 individuals.^7^ MEN1 is estimated to be the cause of 1% to 18% of primary hyperparathyroidism, 16% to 38% of gastrinomas, and up to 3% of pituitary adenomas.^1^ The disease affects all age groups, with a reported range of 5 to 80 years.^8^ While MEN1 is typically inherited, up to 10% of cases are due to de novo MEN1 gene mutations, confirmed via molecular genetic testing.^9^

The diagnostic criteria for MEN1 can be divided into 3 categories: clinical, familial, and genotype-positive MEN1. A diagnosis may be established by one of the 3 criteria, which are described in order as follows: the occurrence of 2 or more primary MEN1-associated endocrine tumors (ie, parathyroid adenoma, enteropancreatic tumor, and pituitary adenoma) in the absence of genetic confirmation; the presence of an MEN1-associated tumor in a patient who has a first-degree relative with MEN1; and the identification of a pathogenic or likely pathogenic germline MEN1 gene variant leading to loss of function of the menin protein, regardless of tumor presence.^10^ Due to its rarity and varied presentation, MEN1 is frequently underdiagnosed or misdiagnosed in clinical settings.

Despite the significant improvement of medical and surgical therapeutic options since the syndrome’s first recognition 60 years ago, the life expectancy of patients with MEN1 is still significantly reduced compared to the general population.^11^ Causes of death in patients with MEN1 can be classified as either MEN-1 related or not. Case series have reported wide variability in non-MEN1-related mortality, ranging from 0% to 72%.^12^ A pooled analysis by Ito et al^12^ of 13 case series revealed a mean of 33% ± 7% of deaths were due to non-MEN1-related causes. In a prospective study conducted at the National Institutes of Health involving 106 patients with MEN1, cardiovascular disease emerged as the leading non-MEN1-related cause of death (accounting for 20% of such deaths), followed by non-MEN1-related malignancies and cerebrovascular disease.^13^ These findings raise the question of whether patients with MEN1 possess an inherently increased risk of cardiovascular disease. Van Wick et al^14^ have suggested this may indeed be the case.

Several cardiovascular risk factors are prevalent among patients with MEN1, particularly hyperparathyroidism and insulin resistance. Both hyperparathyroidism and resultant hypercalcemia have been associated with increased cardiovascular morbidity and mortality.^15^^,^^16^ Additionally, multiple studies have identified a higher prevalence of insulin resistance in patients with MEN1.^14^^,^^17^^,^^18^

In this systematic review and meta-analysis, we aim to further evaluate the extent of insulin resistance among individuals with MEN1. A more profound understanding of cardiovascular risk in this population may provide opportunities to reduce non-MEN1-related mortality and improve long-term outcomes for affected patients.

## Methods

This systematic review and meta-analysis were conducted following the Meta-analysis of Observational Studies in Epidemiology checklist^19^ to ensure a thorough and transparent synthesis of the existing evidence regarding insulin resistance in patients with MEN1. Meta-analysis of Observational Studies in Epidemiology checklist was provided as a supplementary material.

### Search Strategy and Information Sources

A comprehensive literature search was conducted across the following electronic databases: PubMed, Embase, Scopus, Web of Science Core Collection, Cochrane from database inception to February 5, 2025. The search strategy incorporated both controlled vocabulary terms and relevant keywords tailored to each database. Due to resource constraints, the search was restricted to studies published in English. No limitations were applied regarding study design or publication date. Duplicate records were identified and removed using the "Find Duplicates" function in EndNote, followed by manual verification to ensure accuracy. Detailed search strategies for each database are available in the Appendix.

### Eligibility Criteria and Study Selection Process

Studies were eligible for inclusion if they involved adult patients diagnosed with MEN1 and reported measures of insulin resistance. Insulin resistance was defined as diagnosis of prediabetes, type 2 diabetes mellitus, impaired fasting glucose, abnormal oral glucose tolerance test. All study designs were considered, including cohort studies, clinical trials, cross-sectional studies, case-control studies, and case series, provided they were published in English. To ensure sufficient data quality and relevance, studies were required to include a minimum of 5 patients with MEN1. Exclusion criteria included the wrong patient population, populations that included insulinoma or glucagonoma, incorrect diagnoses, non-adult patient populations, insufficient data, and publications not in English. Two independent reviewers initially screened the titles and abstracts of all identified studies for relevance. Full-text articles were retrieved for studies considered potentially relevant, and a second round of independent review was conducted using the predefined inclusion and exclusion criteria. Any disagreements between the reviewers were resolved through discussion or, when necessary, by consulting a third reviewer.

### Data Collection Process and Data Items

Once the final set of studies was determined, data extraction was done by 2 independent reviewers blindly, using a standardized form developed prior to data collection. Extracted data were cross-checked for accuracy, and discrepancies were resolved by discussion and consensus. The data items collected included study characteristics (first author, year of publication, country of origin, study design, and total sample size), demographic information such as age and sex, body mass index (BMI), diagnosis prediabetes or type 2 diabetes mellitus, and Homeostatic Model Assessment of Insulin Resistance (HOMA-IR) index where available. All extracted data were managed and organized using Microsoft Excel prior to statistical analysis and synthesis. All data generated or analyzed during this study are included in this published article and the Supplementary Materials, including the data extraction forms, synthesized data tables, and bias assessment forms and tables. Further information may be provided from the corresponding author upon reasonable request.

### Risk of Bias Assessment

Risk of bias assessment was independently conducted by 2 independent reviewers with each reviewer evaluating all included studies. Any disagreements were resolved through discussion and consensus among the reviewers. To ensure appropriate evaluation based on study design, we used validated, design-specific tools recommended by the Joanna Briggs Institute (JBI)^20^ and the Newcastle–Ottawa Scale (NOS).^21^^,^^22^ For cohort and case-control studies, NOS was utilized, which assesses the methodological quality of studies across 3 domains: the selection of study groups, the comparability of groups, and the ascertainment of exposure or outcome. NOS scores range from 0 to 9, with studies scoring 7–9 considered at low risk of bias, 4–6 as moderate risk, and 0–3 as high risk.^21^ For cross-sectional studies, JBI Critical Appraisal Checklist was used to evaluate methodological aspects such as study objectives, appropriateness of inclusion criteria, reliability measurements, and completeness of data reporting.^20^ JBI checklist for cross-sectional studies includes 8 questions for cross-sectional studies. The studies achieving ≥75% of total possible points were classified as low risk of bias, 50% to 74% as moderate risk, and <50% as high risk. Bias assessments were performed on all included studies. The results of bias assessment were organized into a table incorporating study design, total score, and risk of bias category for each study and included in the Results section.

### Effect Measures and Synthesis Methods

We conducted meta-analyses using a random-effects model (DerSimonian and Laird method) to account for anticipated heterogeneity among studies due to differences in population characteristics, study design, and measurement methods.^22^ The primary outcome was the prevalence of insulin resistance among MEN1 patients compared to controls. Secondary outcome was HOMA-IR index. For insulin resistance, we used odds ratio (OR) to measure the effect size and used Mantel-Hanzel to estimate the pooled effect size. Heterogeneity of studies was assessed using Paul-Mandel method to estimate Tau-squared. For HOMA-IR index, we used standardized mean difference (SMD) to measure the effect size and used Hedges method to estimate the pooled effect size. Heterogeneity of studies was assessed using Restricted Maximum Likelihood method to estimate Tau-squared. Additionally, because there was significant heterogeneity, we used the Knapp-Hartung adjustments to calculate the confidence interval around pooled effect. When standard deviations were not directly reported, they were estimated from available summary statistics, such as ranges or interquartile ranges, using validated formulas.^23^ All statistical analyses were conducted using R version 4.4.1. Specifically, the Meta (Version 8.1-0) and Metafor (Version 4.8-0), The R Foundation packages were used for the meta-analyses.

### Assessment of Heterogeneity, Sensitivity, and Reporting Bias

To assess the robustness of the primary outcome, leave-one-out sensitivity analyses were performed by recalculating the pooled OR each time a single study was removed from the dataset. These results were presented in tabular form to demonstrate the stability of the overall effect estimate. Due to the limited number of studies included in the meta-analysis (*n* = 5), formal statistical tests for publication bias, such as funnel plots or Egger’s regression test, and subgroup analyses were not performed, in accordance with established guidelines.^24^

### Certainty Assessment

The certainty of evidence for the primary outcome (rate of insulin resistance in patients with MEN1 vs control groups) was evaluated using the Grading of Recommendations Assessment, Development and Evaluation (GRADE) framework.^25^ Four out of 5 key domains were assessed including risk of bias, inconsistency, indirectness, imprecision. Publication bias was not assessed due to inadequate number of included studies. Based on this framework, the overall certainty of evidence was rated as high, moderate, low, or very low. The results of the GRADE assessment are summarized in the Results section.

### Registration and Deviations from the Protocol

The review protocol was registered on PROSPERO (CRD420251041613) to ensure transparency and adherence to predefined methodologic standards. Although our systematic review closely followed the methodology outlined in our registered PROSPERO protocol, certain deviations were necessary due to practical considerations encountered during the study.

## Results

### Study Selection

The initial systematic literature review identified a total of 2366 studies across 5 databases: PubMed (*n* = 327), Embase (*n* = 873), Scopus (*n* = 977), Web of Science (*n* = 175), and Cochrane Central Register of Controlled Trials (CENTRAL) (*n* = 14). After removing 939 duplicate studies via EndNote and manual deduplication, 1427 unique studies remained for screening. Titles and abstracts of remaining studies were screened, resulting in 1414 exclusions based on irrelevance or failure to meet inclusion criteria. The full texts of the remaining 13 studies were evaluated for eligibility and resulted in the exclusion of 8 studies for reasons including wrong patient population^26^^,^^27^^,^^28^^,^^29^^,^^30^ wrong study design,^31^ insufficient data.^32^^,^^33^ Ultimately, 5 studies met the inclusion criteria and were included in the qualitative synthesis and quantitative meta-analysis.^14^^,^^17^^,^^18^^,^^34^^,^^35^ The results of the study selection process are illustrated in the Preferred Reporting Items for Systematic reviews and Meta-Analyses flow diagram in Fig.

**Fig:**
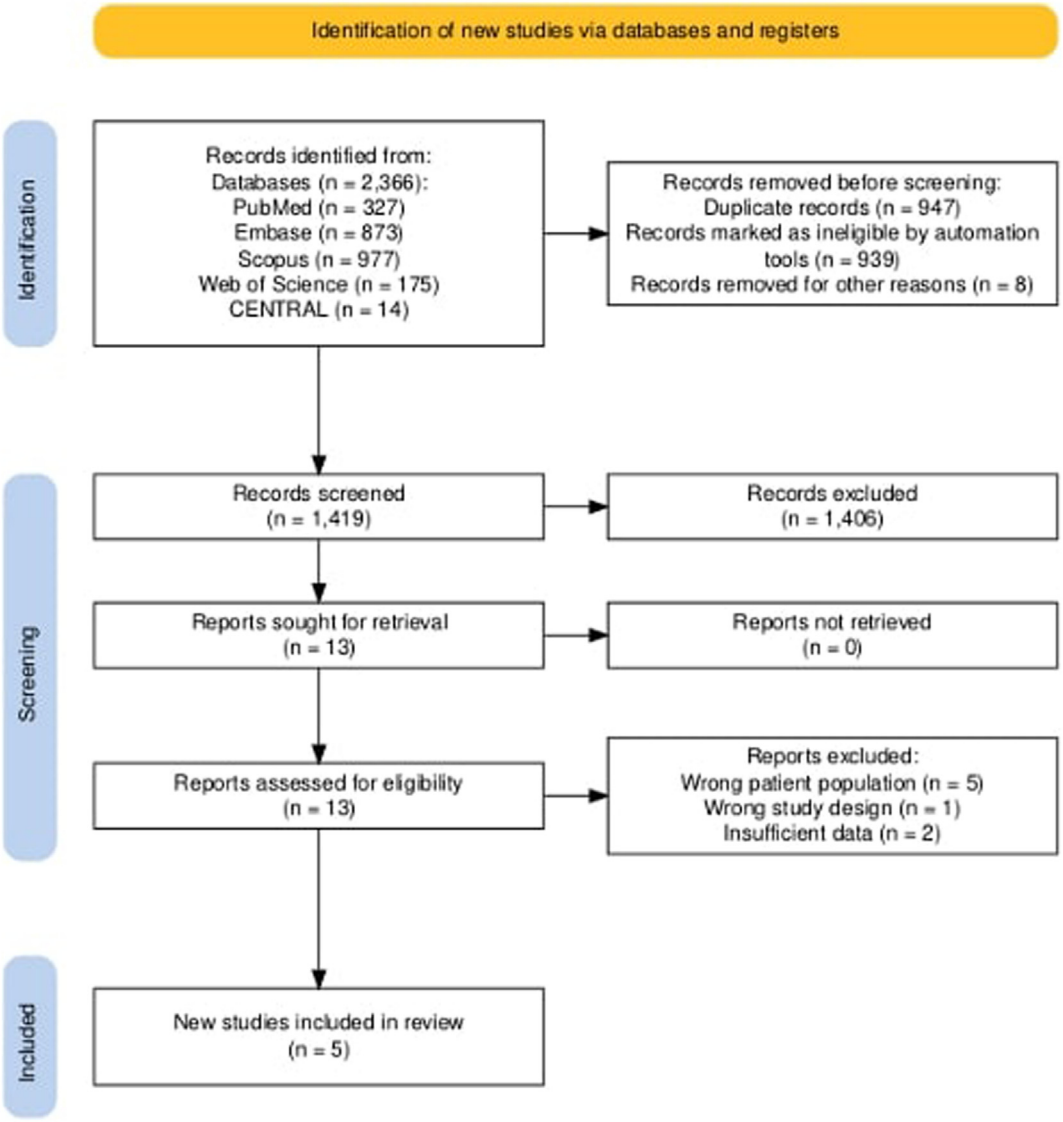
PRISMA flow diagram illustrating the study selection process.

### Study Characteristics

A total of 5 studies, published between 2005 and 2024, were included in the meta-analysis, comprising 606 patients with MEN1 and 4431 controls, as shown in Table 1. The studies varied in design (cross-sectional, case-control, and retrospective cohort) and were conducted in Europe, Asia, Australia, and North America.

**Table 1:** Characteristics of Included Studies

Study	Year	Location	Study design	Sample size			Primary outcome
				Total	MEN1	Control	
Van Wijk et al^14^	2012	Netherlands	Cross-sectional	189	63	126	HOMA-IR index
McCallum et al^17^	2006	Spain	Case-control	205	72	133	Rate of DM and IFG
Wägner et al^18^	2005	Australia	Cross-sectional	31	19	22	Rate of IR
Kim et al^34^	2024	South Korea	Retrospective cohort	4532	412	4120	Rate of DM
Bansal et al^35^	2020	USA	Case-control	80	40	40	Rate of IFG

Abbreviations: DM = diabetes mellitus; HOMA-IR = Homeostatic Model Assessment of Insulin Resistance; IFG = impaired fasting glucose; IR = insulin resistance; MEN1 = multiple endocrine neoplasia type 1.

### Risk of Bias in Studies

None of the included studies were categorized as high risk of bias. Three studies^14^^,^^17^^,^^18^ received full scores (8/8) on the JBI checklist, indicating low risk of bias. These studies clearly described their study populations, applied consistent inclusion criteria, used valid methods for measuring exposures and outcomes, and presented complete outcome data. One retrospective cohort study^34^ was assessed using the NOS and received a score of 6 out of 9, resulting in a moderate risk of bias. Although it had adequate selection and comparability components, it lacked clarity on blinding of outcome assessment and duration of follow-up. One case-control study^35^ also received a score of 6 out of 9 on the NOS, indicating a moderate risk of bias due to concerns regarding the unclear selection of the control group and the absence of a description of exposure ascertainment. Results of risk of bias assessment were illustrated in Table 2.

**Table 2:** Risk of Bias Assessment

Study	Study design	Assessment tool	Score	Risk of bias	Comment
Van Wijk et al^15^	Cross-sectional	JBI	8/8	Low	High methodologic quality
McCallum et al^18^	Cross-sectional	JBI	7/8	Low	Confounding factors were not addressed
Wägner et al^19^	Cross-sectional	JBI	6/8	Low	Study settings and criteria for insulin resistance were not described
Kim et al^35^	Retrospective cohort	NOS	6/9	Moderate	Loss to follow-up and assessment of outcome were not described
Bansal et al^36^	Case-control	NOS	6/9	Moderate	Selection of controls and ascertainment of exposure were not described

Abbreviations: JBI = Joanna Briggs Institute; NOS = Newcastle–Ottawa Scale.

### Results of Individual Studies and Synthesis

The mean age was comparable between MEN1 and control groups (43.28 ± 13.64 vs43.00 ± 13.38 years, respectively; *P* = 0.687), as was the proportion of male participants (37% vs36%; *P* = 0.784). Similarly, mean BMI values did not differ significantly between groups (26.32 ± 5.53 kg/m^2^ vs26.64 ± 6.44 kg/m^2^; *P* = 0.356). Results of individual studies are summarized in Table 3.

**Table 3:** Results of Individual Studies

Study	Sample size			Male sex (%)		Age		BMI (kg/m^2^)		IR (*n*)		HOMA-IR	
	Total	MEN1	Control	MEN1	Control	MEN1	Control	MEN1	Control	MEN1	Control	MEN1	Control
Van Wijk et al^14^	189	63	126	44	44	41 ± 11.75^a^	41 ± 11.50^a^	26.3 ± 4.9	26.2 ± 4.7	14	11	3 ± 2	2 ± 1
McCallum et al^17^	205	72	133	35	32	44.06 ± 12.72	41.45 ± 13.05	NS	NS	19	9	3.76 ± 1.87^b^	2.18 ± 0.43^c^
Wägner et al^18^	31	19	22	47	58	41.7 ± 15.2	35.93 ± 14.46^d^	23.17 ± 5.11^d^	28.73 ± 14.70^d^	6	0	2.22 ± 4.38^e^	1.67 ± 1.13^e^
Kim et al^34^	4532	412	4120	35	35	43.6 ± 15.8	43.6 ± 15.8	NS	NS	93	198	NS	NS
Bansal et al^35^	80	40	40	NS	NS	41 ± 11	41 ± 11	29.2 ± 7.2	29.1 ± 7.5	22	4	4.01 ± 0.61^f^	2.44 ± 0.37^f^

Abbreviations: BMI = body mass index; HOMA-IR = Homeostatic Model Assessment of Insulin Resistance; IQR = interquartile range; IR = insulin resistance; MEN1 = multiple endocrine neoplasia type 1; NS = not stated; SD = standard deviation.

Age, BMI, HOMA-IR index reported as mean ± SD.

a Reported as mean and range by writers. Data converted to mean and SD by using validated formulas.^23^

b HOMA-IR index calculated for 58 patients with MEN1.

c HOMA-IR index calculated for 114 controls.

d Reported as median and IQR by writers. Data converted to mean and SD by using validated formulas.^23^

e HOMA-IR index was calculated from reported fasting glucose and insulin levels.

f Reported as mean without SD by writers. SD was estimated.

Meta-analyses were conducted for the predefined outcomes, and the findings are presented in Table 4. The primary analysis, evaluating the OR for the prevalence of insulin resistance, included all 5 studies.^14^^,^^17^^,^^18^^,^^34^^,^^35^ Insulin resistance was significantly more common in the MEN1 group with an OR of 5.58 (95% confidence interval (CI)) [4.02, 7.74]. No significant heterogeneity was observed (Tau^2^ = 0, *P* = 0.4552). The secondary analysis, evaluating the SMD of HOMA-IR index, was included by 4 of the 5 studies.^14^^,^^17^^,^^18^^,^^35^ There was no statistically significant difference of HOMA-IR index between groups with SMD of 1.33 (95% CI [−0.67, 1.33]). Heterogeneity was high (Tau2 = 1.4685, *P* < 0.0001).

**Table 4:** Results of Syntheses

Study	Weight	IR OR [95% CI]	Weight	HOMA-IR SMD [95% CI]
Van Wijk et al^14^	8%	2.99 [1.27, 7.04]	25.8%	0.71 [0.39, 1.02]
McCallum et al^17^	8%	4.94 [2.1, 11.62]	25.7%	1.38 [1.03, 1.73]
Wägner et al^18^	0.7%	12.04 [0.61, 236.4]	24%	0.15 [-0.57, 0.88]
Kim et al^34^	79.4%	5.77 [4.4, 7.58]	NS	NS
Bansal et al^35^	4%	11 [3.29, 36.75]	24.4%	3.08 [2.43, 3.74]
Total	100%	5.58 [4.02, 7.74]	100%	1.33 [-0.67, 3.33]

Abbreviations: CI = confidence interval; HOMA-IR = Homeostatic Model Assessment of Insulin Resistance; IR = insulin resistance; NS = not stated; OR = odds ratio; SMD = standardized mean difference.

### Sensitivity Analyses

To assess the robustness of the primary findings, we performed leave-one-out sensitivity analyses for the primary and secondary outcomes. When we excluded each of all 5 studies in turn, the recalculated OR for insulin resistance remained consistently elevated, ranging from 4.98 to 5.89. The OR consistently remained statistically significant (*P* < 0.05) with only minimal variations, indicating that no single study disproportionately influenced the overall effect size. The results of the sensitivity analysis for insulin resistance are presented in Table 5. Similarly, the leave-one-out analysis for HOMA-IR index showed not statistically significant (*P* > 0.05) results with SMD ranging from 0.8 to 1.7. CI consistently included zero with each analysis. The sensitivity analyses confirm the robustness and reliability of the primary meta-analysis results for rate of insulin resistance.

**Table 5:** Leave-One-Out Analysis for Insulin Resistance

Omitted study	Sample size	OR	95% CI	*P* - value
Van Wijk et al^14^	4848	5.89	4.44, 7.82	<0.001
McCallum et al^17^	4832	5.54	2.83, 10.84	0.004
Wägner et al^18^	5006	5.42	3.13, 9.36	0.002
Kim et al^34^	505	4.98	1.96, 12.64	0.012
Bansal et al^35^	4957	5.42	3.79, 7.76	0.001

Abbreviations: CI = confidence interval; OR = odds ratio.

### Certainty of Evidence

We assessed the certainty of evidence for the primary outcome, the prevalence of insulin resistance, using the GRADE approach. Based on data from 5 observational studies, the certainty was rated as moderate. The evidence was not downgraded for risk of bias, inconsistency, indirectness, or imprecision. There was no serious risk of bias, and heterogeneity was low (I^2^ = 0%), indicating consistent findings across studies. The outcome was considered direct and precise and demonstrated a large effect size (OR = 5.58, 95% CI [4.02, 7.74]). However, because the body of evidence was based on observational studies, the initial GRADE level was low and could only be upgraded by one level. The assessment was limited due to the absence of a publication bias evaluation. Nevertheless, the evidence indicates a moderately high level of confidence that insulin resistance is more prevalent among patients with MEN1 compared to controls.

## Discussion

Our meta-analysis demonstrates that the prevalence of insulin resistance is significantly higher in patients with MEN1 compared to controls. Several pathophysiological mechanisms may contribute to this association, particularly comorbid conditions commonly seen in MEN1, such as hypercalcemia and pancreatic neuroendocrine tumors. Evidence from prior studies supports the role of calcium metabolism in glucose homeostasis. For example, Becerra-Thomas et al^36^ conducted a prospective cohort study and found a positive correlation between albumin-adjusted serum calcium levels and the prevalence of type 2 diabetes mellitus. Calcium levels affect glucose homeostasis at multiple levels. Insulin release from pancreatic β-cells in response to hyperglycemia is a calcium-dependent process mediated by voltage-gated calcium channels.^37^ Elevated extracellular calcium concentrations can disrupt these channels, impairing insulin secretion.^38^ Additionally, calcium influences the expression and function of glucose transporter type 4 (GLUT4). In skeletal muscle cells, altered calcium levels can downregulate GLUT4 expression,^39^ and in adipocytes, hypercalcemia may reduce insulin sensitivity and the number of GLUT4 transporters.^40^

Unfortunately, serum calcium and parathyroid hormone levels were not included in our meta-analysis due to inconsistencies in data reporting across the included studies. However, several included studies provide further insight. McCallum et al^17^ reported that patients with MEN1 and hyperparathyroidism had a significantly higher prevalence of insulin resistance compared to those with MEN1 and normocalcemia (75% vs 32.5%, *P* < 0.01). Similarly, Wagner et al^18^ found that HOMA-IR index was significantly elevated in MEN1 patients with hyperparathyroidism compared to those without. In contrast, van Wijk et al^14^ observed no significant association between HOMA-IR and either serum calcium levels or the presence of hyperparathyroidism in their MEN1 cohort.

McCallum et al^17^ speculated that hormones secreted by pancreatic neuroendocrine tumors (PNETs) may contribute to insulin resistance in patients with MEN1. In their study, serum gastrin levels were significantly elevated in the MEN1 group compared to controls (50% vs 20.9%, *P* < 0.05), and chromogranin A levels approached statistical significance (*P* = 0.056). Based on these findings, the authors proposed that gastrin and chromogranin A might serve as surrogate markers for unidentified cytokines or paracrine factors involved in the pathophysiology of insulin resistance. However, this hypothesis is not uniformly supported. For example, Wagner et al^18^ reported that neither serum gastrin levels nor the presence of PNETs significantly influenced HOMA-IR values. Overall, these findings raise the possibility that insulin resistance in MEN1 may not be solely attributable to secondary metabolic disturbances such as hypercalcemia and the presence of a potential intrinsic metabolic risk associated with MEN1 syndrome.

In addition to metabolic factors such as hypercalcemia, genetic mechanisms may also contribute to the increased insulin resistance observed in MEN1. Menin, the product of the *MEN1* gene, is a coactivator for PPARγ, a transcription factor critical for adipocyte differentiation and the expression of genes that enhance insulin sensitivity.^3^ Disruption of this pathway may impair adipocyte function and contribute to systemic insulin resistance. In a recent animal study, Liu et al demonstrated that menin plays a direct role in regulating hepatic glucose uptake, further implicating *MEN1* dysfunction in the pathogenesis of insulin resistance.^41^ Additionally, menin has been shown to influence insulin secretion from pancreatic β-cells, suggesting its involvement at multiple levels of glucose metabolism.^42^ These findings support the hypothesis that *MEN1* mutations may independently contribute to the development of insulin resistance, beyond the effects of associated endocrine tumors or metabolic complications.

The lack of a statistically significant difference in the HOMA-IR index between groups, despite a markedly higher prevalence of insulin resistance in MEN1, likely reflects methodological variability. The assessment of the HOMA-IR index varied considerably across the included studies, leading to substantial heterogeneity. Differences included the method of reporting (mean vs median), whether standard deviations were provided or imputed, and whether HOMA-IR was derived from fasting glucose and insulin values or directly reported. Furthermore, the prevalence of insulin resistance is a binary measure, which can reveal clinically meaningful differences that may be obscured when HOMA-IR is analyzed as a continuous variable. Together, these factors likely explain why the prevalence of insulin resistance was significantly elevated in MEN1 patients, while the analysis of the HOMA-IR index failed to demonstrate a significant difference, with considerable heterogeneity.

Our study has several limitations. First, the literature search was restricted to articles published in English, which may have introduced language bias by excluding potentially relevant studies published in other languages. Second, considerable heterogeneity was observed across the included studies in terms of study design, sample size, and reporting of key confounding variables such as the presence of PNETs, hyperparathyroidism, and hypercalcemia. These variations limited our ability to conduct more refined subgroup analyses and may have contributed to residual confounding. Third, the small number of studies (*n* = 5) included in this meta-analysis reduced statistical power and limited the robustness of our results. Fourth, the small number of included studies also restricted our capacity to formally assess publication bias through funnel plots or statistical tests. As a result, the possibility of publication bias cannot be excluded, and the prevalence of insulin resistance among patients with MEN1 may be subject to overestimation. Fifth, not all data used in our meta-analysis were reported directly in the original publications. For example, van Wijk et al^14^ reported age using mean and range, and Wagner et al^18^ presented BMI as median and interquartile range. In these cases, estimates of standard deviation were calculated using established statistical methods. Furthermore, HOMA-IR values in Wagner et al^18^ were derived from reported fasting glucose and insulin levels, and Bansal et al^35^ did not provide standard deviation for HOMA-IR, which was subsequently imputed based on available data. These necessary conversions introduced potential estimation error, which should be considered when interpreting the results.

Additionally, the studies included in the meta-analysis also have several limitations that may influence the interpretation of our findings. All 5 studies^14^^,^^17^^,^^18^^,^^34^^,^^35^ employed observational designs; no prospective cohort studies or randomized controlled trials were identified during our systematic search. The observational nature of these studies introduces inherent risks of selection and measurement biases. Specifically, the lack of randomization and blinding increases the likelihood of confounding.

Notably, 68% of the MEN1 cohort originated from a single study.^34^ To assess the robustness of our primary outcome, we performed a leave-one-out sensitivity analysis. The exclusion of any individual study did not materially affect the pooled effect estimates or their statistical significance, indicating that the findings were not disproportionately influenced by any single study. Heterogeneity among studies reporting insulin resistance prevalence was low (I^2^ = 0), supporting the consistency of the observed effect.

To our knowledge, this is the first systematic review and meta-analysis to examine the association between insulin resistance and MEN1 syndrome. Our findings demonstrate that patients with MEN1 exhibit a significantly higher prevalence of insulin resistance compared to controls. This metabolic disturbance is likely a key contributor to the increased cardiovascular mortality observed in this patient group. Although hypercalcemia is a well-established factor in the pathogenesis of insulin resistance, the studies included in our analysis demonstrated inconsistent results regarding its role, suggesting that additional underlying mechanisms may be involved. One such possibility is the disruption of MEN1 gene function, which has been implicated in the regulation of glucose metabolism and insulin signaling pathways.

As advancements in the management of MEN1-associated tumors continue to improve survival, cardiovascular disease is expected to become an increasingly prominent cause of morbidity and mortality in this population. Therefore, we recommend that patients with MEN1 be screened for insulin resistance early in the disease course, which may help mitigate long-term cardiovascular risks and improve life expectancy. Further research is warranted to elucidate the precise molecular mechanisms linking MEN1 to insulin resistance and to conduct subgroup analyses that clarify the associations of hyperparathyroidism and PNETs with insulin resistance in patients with MEN1.

## Declaration of Generative AI and AI-Assisted Technologies in the Writing Process

During the preparation of this work, the author used ChatGPT (OpenAI) to improve the clarity, grammar, and consistency of the English language. Following the use of this tool, the author carefully reviewed, edited, and verified the content to ensure accuracy and takes full responsibility for the final version of the manuscript.

## Disclosure

Dr Michael Brennan is a paid consultant for Novo Nordisk, Boehringer Ingelheim, and Bayer. The rest of the authors declare that they have no known competing financial interests or personal relationships that could have appeared to influence the work reported in this paper.

## Glossary

Adipocyte: A specialized cell in adipose tissue that primarily stores triglycerides.
Beta cell: A specialized cell in the pancreas responsible for producing and secreting insulin.
Chromogranin A (CgA): A protein found inside neuroendocrine cells, which release CgA and certain hormones into the blood. CgA is a type of tumor marker.
Gastrin: A hormone released from special cells in the lining of the stomach after eating.
Glucose transporter type 4: A protein that facilitate the uptake of glucose in muscle and fat cells, particularly in response to insulin.
Homeostatic Model Assessment of Insulin Resistance: A method used to estimate insulin resistance from fasting glucose and insulin levels.
Menin: A 610-amino acid which encoded by nuclear protein multiple endocrine neoplasia type 1 tumor suppressor gene. Menin participates in a variety of cellular processes, including transcriptional regulation, maintenance of chromosomal integrity, and deoxyribonucleic acid replication.
Multiple endocrine neoplasia type 1: a hereditary disorder characterized by tumors in endocrine glands such as the parathyroid, pancreas, and pituitary.
Neuroendocrine tumor: A tumor that forms from cells that release hormones into the blood in response to a signal from the nervous system. Neuroendocrine tumors may make higher-than-normal amounts of hormones, which can cause many different symptoms.
Oral glucose tolerance test: A glucose tolerance test is a procedure that determines whether a patient can use and store glucose normally.
Peroxisome proliferator-activated receptor gamma: A type of nuclear receptor that functions as a transcription factor. It plays a crucial role in regulating various cellular processes, including adipocyte differentiation, glucose homeostasis, and lipid metabolism.

## Supplementary data

MOOSE Checklist

Search strategy
