# Supplementary material for: Insulin Resistance Among Patients With Multiple Endocrine Neoplasia Type 1: A Systemic Review and Meta-Analysis
Source: AACE Endocrinol Diabetes. 2025 Sep 3;13(1):2–9. doi: 10.1016/j.aed.2025.08.018 (PMC12866170; doi:10.1016/j.aed.2025.08.018)
Supplement: Search strategy [file mmc2.docx]

**Literature Search Strategies**

*PubMed Search Strategy*

#1 “Multiple Endocrine Neoplasia” [Mesh] OR “Multiple Endocrine Neoplasia, Type IV”

[Supplementary Concept]

#2 multiple [tw] AND endocrine [tw] AND (neoplasm* [tw] OR neoplasia* [tw] OR

adenomatos* [tw] OR adenopath* [tw])

#3 familial [tw] AND endocrine [tw] AND (adenomatos* [tw] OR adenopath* [tw])

#4 wermer* [tw] OR wagenmann* [tw] OR sipple* [tw] AND syndrome [tw]

#5 pheochromocytoma [tw] AND amyloid* [tw] AND medullary [tw] AND thyroid [tw]

AND carcinoma* [tw]

#6 neuromata [tw] AND mucosal [tw] AND endocrine [tw]

#7 mucosal [tw] AND neuroma [tw] AND syndrome* [tw]

#8 men1 [tw] OR men2 [tw] OR men2a [tw] OR men2b [tw] OR men4 [tw] OR menIV

[tw] OR menIIa [tw] OR menIIb [tw] OR menI [tw]

#9 #1 OR #2 OR #3 OR #4 OR #5 OR #6 OR #7 OR #8

#10 “Diabetes Mellitus” [Mesh] OR “Insulin Resistance” [Mesh]

#11 diabetes [tw] OR diabetic* [tw] OR prediabet* [tw]

#12 “insulin resistance” OR “insulin sensitivity” OR “metabolic syndrome*” OR

“cardiometabolic syndrome*” OR “dysmetabolic syndrome*”

#13 #10 OR #11 OR #12

#14 #9 AND #13

#15 #9 AND #13 Filters: English

*Embase Search Strategy*

#1 ‘multiple endocrine neoplasia’/exp

#2 multiple:ti,ab,kw AND endocrine:ti,ab,kw AND (neoplasm*:ti,ab,kw OR

neoplasia*:ti,ab,kw OR adenomatos*:ti,ab,kw OR adenopath*:ti,ab,kw)

#3 familial:ti,ab,kw AND endocrine:ti,ab,kw AND (adenomatos*:ti,ab,kw OR

adenopath*:ti,ab,kw)

#4 wermer*:ti,ab,kw OR sipple*:ti,ab,kw OR wagenmann*:ti,ab,kw AND

syndrome:ti,ab,kw

#5 pheochromocytoma:ti,ab,kw AND amyloid*:ti,ab,kw AND medullary:ti,ab,kw AND

thyroid:ti,ab,kw AND carcinoma*:ti,ab,kw

#6 neuromata*:ti,ab,kw AND mucosal:ti,ab,kw AND endocrine:ti,ab,kw

#7 mucosal:ti,ab,kw AND neuroma:ti,ab,kw AND syndrome*:ti,ab,kw

#8 men1:ti,ab,kw OR meni:ti,ab,kw OR men2:ti,ab,kw OR menii:ti,ab,kw OR

men2a:ti,ab,kw OR meniia:ti,ab,kw OR men2b:ti,ab,kw OR meniib:ti,ab,kw OR

men4:ti,ab,kw OR meniv:ti,ab,kw

#9 #1 OR #2 OR #3 OR #4 OR #5 OR #6 OR #7 OR #8

#10 ‘diabetes mellitus’/exp OR ‘insulin resistance’/exp OR ‘insulin sensitivity’/exp OR ‘metabolic syndrome x’/exp

#11 diabetes:ti,ab,kw OR diabetic*:ti,ab,kw OR prediabet*:ti,ab,kw

#12 ‘insulin resistance’:ti,ab,kw OR ‘insulin sensitivity’:ti,ab,kw OR ‘metabolic

syndrome*’:ti,ab,kw OR ‘metabolic x syndrome*’:ti,ab,kw OR ‘cardiometabolic

syndrome*’:ti,ab,kw OR ‘dysmetabolic syndrome*’:ti,ab,kw

#13 #10 OR #11 OR #12

#14 #9 AND #13

#15 #9 AND #13 AND [English]/lim

*Scopus Search Strategy*

#1 TITLE-ABS-KEY(multiple) AND TITLE-ABS-KEY(endocrine) AND TITLE-ABS-KEY(

neoplasm* OR neoplasia* OR adenomatos* OR adenopath*)

#2 TITLE-ABS-KEY(familial) AND TITLE-ABS-KEY(endocrine) AND TITLE-ABS-KEY(

adenomatos* OR adenopath*)

#3 TITLE-ABS-KEY(wermer* OR sipple* OR wagenmann*) AND TITLE-ABS-

KEY(syndrome)

#4 TITLE-ABS-KEY(pheochromocytoma AND amyloid* AND medullary AND thyroid

AND carcinoma*)

#5 TITLE-ABS-KEY(neuromata AND mucosal AND endocrine)

#6 TITLE-ABS-KEY(mucosal AND neuroma AND syndrome*)

#7 TITLE-ABS-KEY(men1 OR meni OR men2 OR menii OR men2a OR meniia OR

men2b OR meniib OR men4 OR meniv)

#8 #1 OR #2 OR #3 OR #4 OR #5 OR #6 OR #7

#9 TITLE-ABS-KEY(diabetes OR diabetic* OR prediabet*)

#10 TITLE-ABS-KEY(“insulin resistance”) OR TITLE-ABS-KEY(“insulin sensitivity”) OR

TITLE-ABS-KEY(“metabolic syndrome*”) OR TITLE-ABS-KEY(“metabolic x

syndrome*) OR TITLE-ABS-KEY(“cardiometabolic syndrome*”) OR TITLE-ABS-

KEY(“dysmetabolic syndrome*”)

#11 #9 OR #10

#12 #8 AND #11

#13 #8 AND #11 AND LIMIT-TO (LANGUAGE, “English”)

*Web of Science Search Strategy*

#1 TS=(multiple AND endocrine AND (neoplasm* OR neoplasia* OR adenomatos* OR

adenopath*))

#2 TS=(familial AND endocrine AND (adenomatos* OR adenopath*))

#3 TS=(wermer* OR sipple* OR wagenmann* AND syndrome)

#4 TS=(pheochromocytoma AND amyloid* AND medullary AND thyroid AND

carcinoma*)

#5 TS=(neuromata AND mucosal AND endocrine)

#6 TS=(mucosal AND neuroma AND syndrome*)

#7 TS=(men1 OR menI OR men2 OR menII OR men2a OR menIIa OR men2b OR

menIIb OR men4 OR menIV)

#8 #1 OR #2 OR #3 OR #4 OR #5 OR #6 OR #7

#9 TS=(diabetes OR diabetic* OR prediabet*)

#10 TS=(“insulin resistance”) OR TS=(“insulin sensitivity”) OR TS=(“metabolic

syndrome*”) OR TS=(“metabolic x syndrome*”) OR TS=(“cardiometabolic

syndrome*”) OR TS=(“dysmetabolic syndrome*”)

#11 #9 OR #10

#12 #8 AND #11

#13 #8 AND #11 AND English (Languages)

*Cochrane Search Strategy*

#1 MeSH descriptor: [Multiple Endorine Neoplasia] explode all tress

#2 (multiple AND endocrine AND (neoplasm* OR neoplasia* OR adenomatos* OR

adenopath*)):ti,ab,kw

#3 (familial AND endocrine AND (adenomatos* OR adenopath*)):ti,ab,kw

#4 (wermer* OR sipple* OR wagenmann* AND (syndrome)):ti,ab,kw

#5 (pheochromocytoma AND amyloid* AND medullary AND thyroid AND carcinoma*):

ti,ab,kw

#6 (neuromata AND mucosal AND endocrine):ti,ab,kw

#7 (mucosal AND neuroma AND syndrome*):ti,ab,kw

#8 (men1 OR menI OR men2 OR menII OR men2a OR menIIa OR men2b OR menIIb

OR men4 OR menIV):ti,ab,kw

#9 #1 OR #2 OR #3 OR #4 OR #5 OR #6 OR #7 OR #8

#10 MeSH descriptor: [Diabetes Mellitus] explode all trees

#11 MeSH descriptor: [Insulin Resistance] explode all trees

#12 (diabetes OR diabetic* OR prediabetic*):ti,ab,kw

#13 (“insulin resistance”):ti,ab,kw

#14 (“insulin sensitivity”):ti,ab,kw

#15 (metabolic NEXT syndrome*):ti,ab,kw

#16 (cardiometabolic NEXT syndrome*):ti,ab,kw

#17 (dysmetabolic NEXT syndrome*):ti,ab,kw

#18 #10 OR #11 OR #12 OR #13 OR #14 OR #15 OR #16 OR #17

#19 #9 AND #18
